# Periprosthetic Joint Infection Caused by Providencia stuartii: A Case Report

**DOI:** 10.7759/cureus.72868

**Published:** 2024-11-02

**Authors:** Atanas Panev, Ivan Ivanov, Georgi P Georgiev, Lyubomir Gaydarski, Plamen Kinov

**Affiliations:** 1 Department of Orthopaedics and Traumatology, University Hospital Queen Giovanna - ISUL, Sofia, BGR; 2 Department of Microbiology, National Center of Infectious and Parasitic Diseases, Sofia, BGR; 3 Department of Anatomy, Histology and Embryology, Medical University of Sofia, Sofia, BGR

**Keywords:** meropenem infusion pump, periprosthetic joint infection, providencia stuartii, spacer exchange, two-stage revision

## Abstract

Periprosthetic joint infection (PJI) is a rare yet serious complication following a total hip replacement, predominantly caused by gram-positive bacteria such as *Staphylococcus aureus* and *Staphylococcus epidermidis. *However, gram-negative pathogens can also be isolated. This case report presents a 22-year-old patient with a PJI caused by *Providencia stuartii *after total hip replacement due to a motor vehicle accident. The patient with a clinical history of total hip replacement secondary to posttraumatic arthrosis presented to our department with clinical and laboratory indications of PJI in the right hip. Microbiological samples revealed *P. stuartii*, a multidrug-resistant gram-negative pathogen. A two-stage revision with a double spacer exchange procedure was performed. Antibiotic treatment with prolonged meropenem infusion via an infusion pump was administered. After two years of follow-up, no signs of infection were observed. The exchange spacer procedure with prolonged antibiotic administration for the treatment of difficult-to-treat pathogens causing PJI is a high-risk procedure associated with significant mortality. Nevertheless, for young patients with robust reparative capabilities, it may be considered a viable therapeutic option.

## Introduction

Periprosthetic joint infection (PJI) following total hip arthroplasty is a severe complication associated with significant morbidity and healthcare expenditures [1]. Moreover, the optimal diagnostic approach and management of these infections remain subjects of debate, for more than a decade scientific societies have been mobilized to find valid criteria to define a PJI. The 2018 Musculoskeletal Infection Society (MSIS)/International Consensus Meeting (ICM) criteria and the 2021 European Bone and Joint Infection Society (EBJIS) criteria are both widely used definitions for diagnosing PJI, each with strong sensitivity and specificity [2,3]. While one or more microorganisms may be responsible for PJI, bacterial infections are the most prevalent. The most frequently isolated bacterium is *Staphylococcus aureus*, accounting for 72% of all cases, whereas gram-negative bacteria constitute a substantial proportion ranging from 5% to 23% [4].

Identifying the causative pathogen is crucial to improve treatment outcomes in PJI management. However, 7-15% of PJIs yield negative cultures, complicating management and increasing reoperation rates [2]. In such instances, empirical broad-spectrum antibiotics are recommended, and advanced molecular techniques such as Sanger and next-generation sequencing (NGS) can be advantageous for detection [5]. However, atypical pathogens such as *Providencia stuartii* are rarely reported in the literature. We present a case report of a 22-year-old patient with chronic PJI caused by *P. stuartii*.

## Case presentation

**Figure 1:**
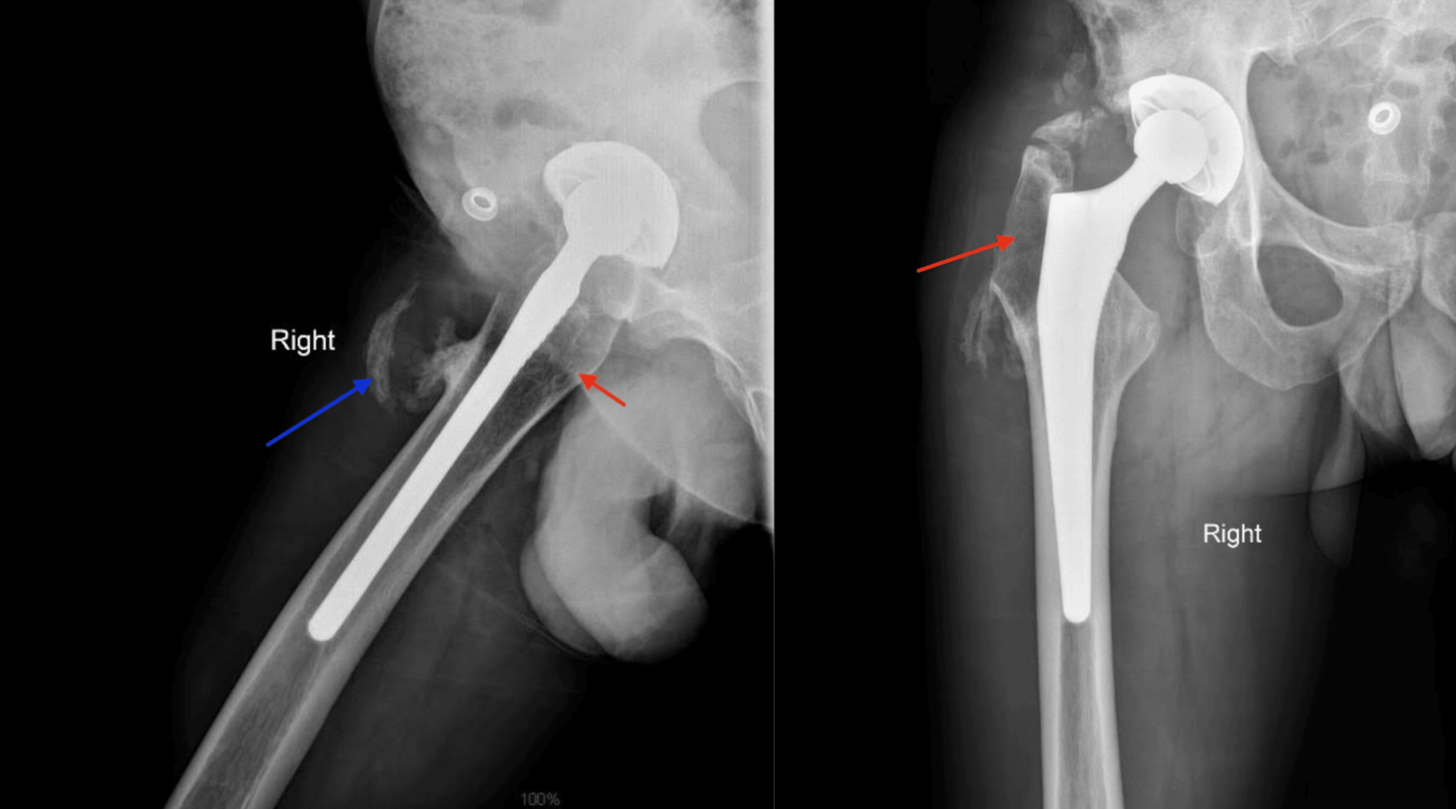
Preoperative AP and lateral X-rays showing osteolysis around the stem fixation area (red arrows) and bone remodeling associated with the fistula (blue arrow).

A 22-year-old male with no medical history of comorbidities and prior surgeries experienced a motor vehicle collision, resulting in a diagnosis of right acetabulum fracture and multiple vertebral fractures. An open reduction and internal fixation of the acetabulum and stabilization of the lumbar spine were performed in emergency settings. Three days postoperatively, a septic condition was observed. Extraction of the plate was performed in conjunction with prolonged antibiotic therapy utilizing broad-spectrum antibiotics. No microbiological study is available from this period. One year subsequently, total hip replacement was performed due to post-traumatic arthrosis. One month postoperatively, the patient presented with swelling, erythema, and pain in the right hip. Multiple soft-tissue debridements and mobile component exchanges were performed. Microbiological analysis of tissue cultures obtained during surgery revealed *P. stuartii*, a gram-negative multidrug-resistant pathogen. However, symptoms persisted, and in July 2020, the patient was admitted to our department with a fistula. A preoperative aspiration with microbiological sampling was performed, which again identified the same pathogen. Upon admission, all laboratory tests indicated inflammation. The white blood cell count and the polymorphonuclear neutrophils percentage (PMN %) were elevated above the established threshold values following joint aspiration. The preoperative radiographs demonstrated heterotopic ossification and osteolysis in the area of stem fixation. On the lateral X-ray, bone remodeling associated with fistula formation was observed (Figure 1).

**Figure 2:**
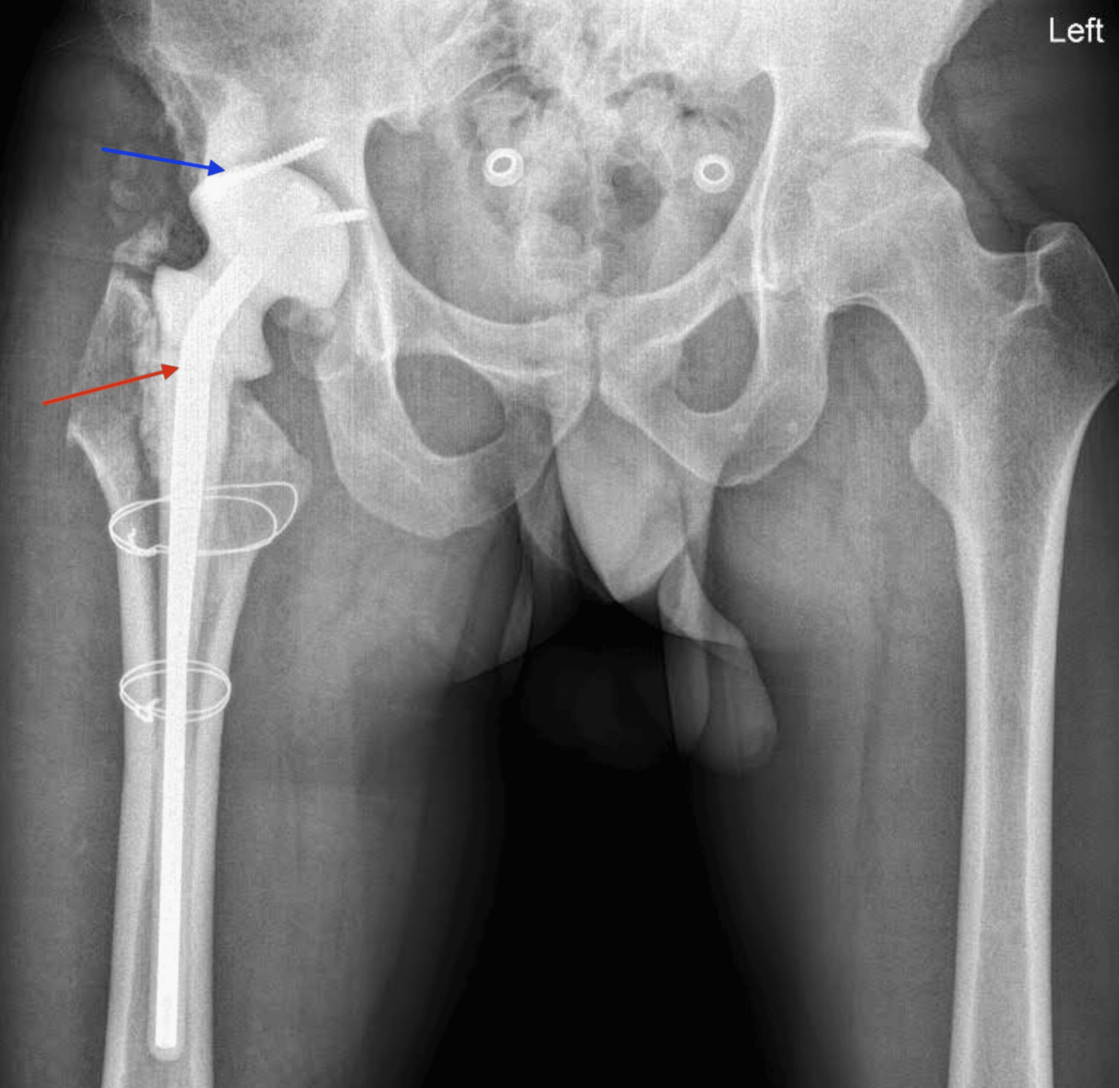
Postoperative X-ray showing bone cement and screw augmentation for luxation prevention (blue arrow) and antibiotic-loaded spacer placement (red arrow).

A decision for a two-stage revision was made. Following implantation of an antibiotic-loaded spacer, augmentation with bone cement (both loaded with vancomycin and gentamicin) and screws were done for luxation prevention. Two weeks of intravenous antibiotic therapy with levofloxacin (500mg) and amikacin (1000mg) at therapeutic doses was administrated and the patient was discharged (Figure 2). Oral levofloxacin was administered for a month following discharge from the hospital.

**Figure 3:**
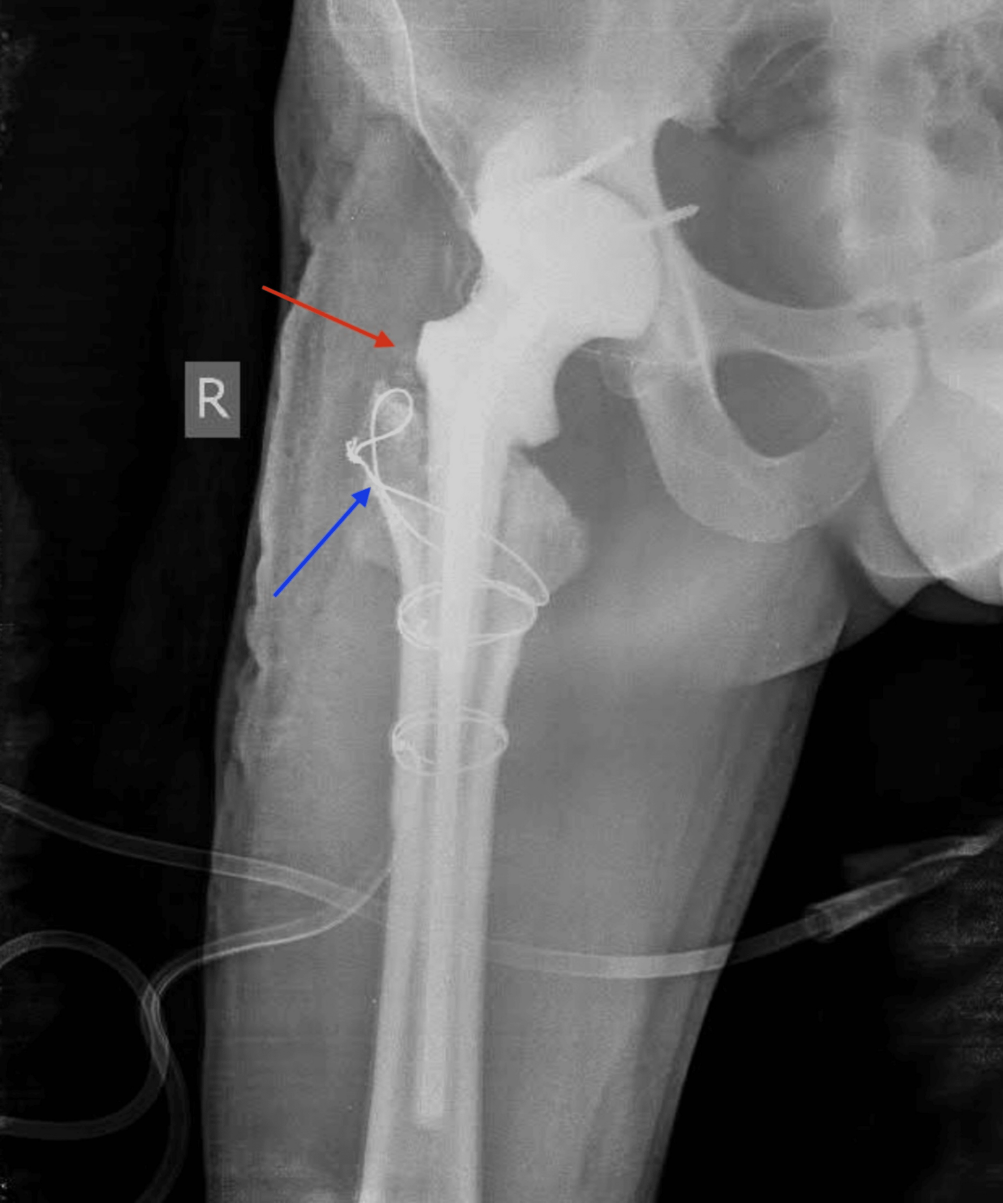
Postoperative X-ray (first spacer exchange) showing trochanteric osteolysis (red arrow) and wire reattachment of the abductors (blue arrow).

Nevertheless, symptoms persisted, and clinical manifestation of infection was observed. A spacer exchange procedure was conducted. During the second revision, extensive trochanteric osteolysis was observed. The abductors were reattached with wires and sutures. Another intravenous antibiotic regimen was initiated, incorporating meropenem (1000mg) administered thrice daily and excluding amikacin (Figure 3).

**Figure 4:**
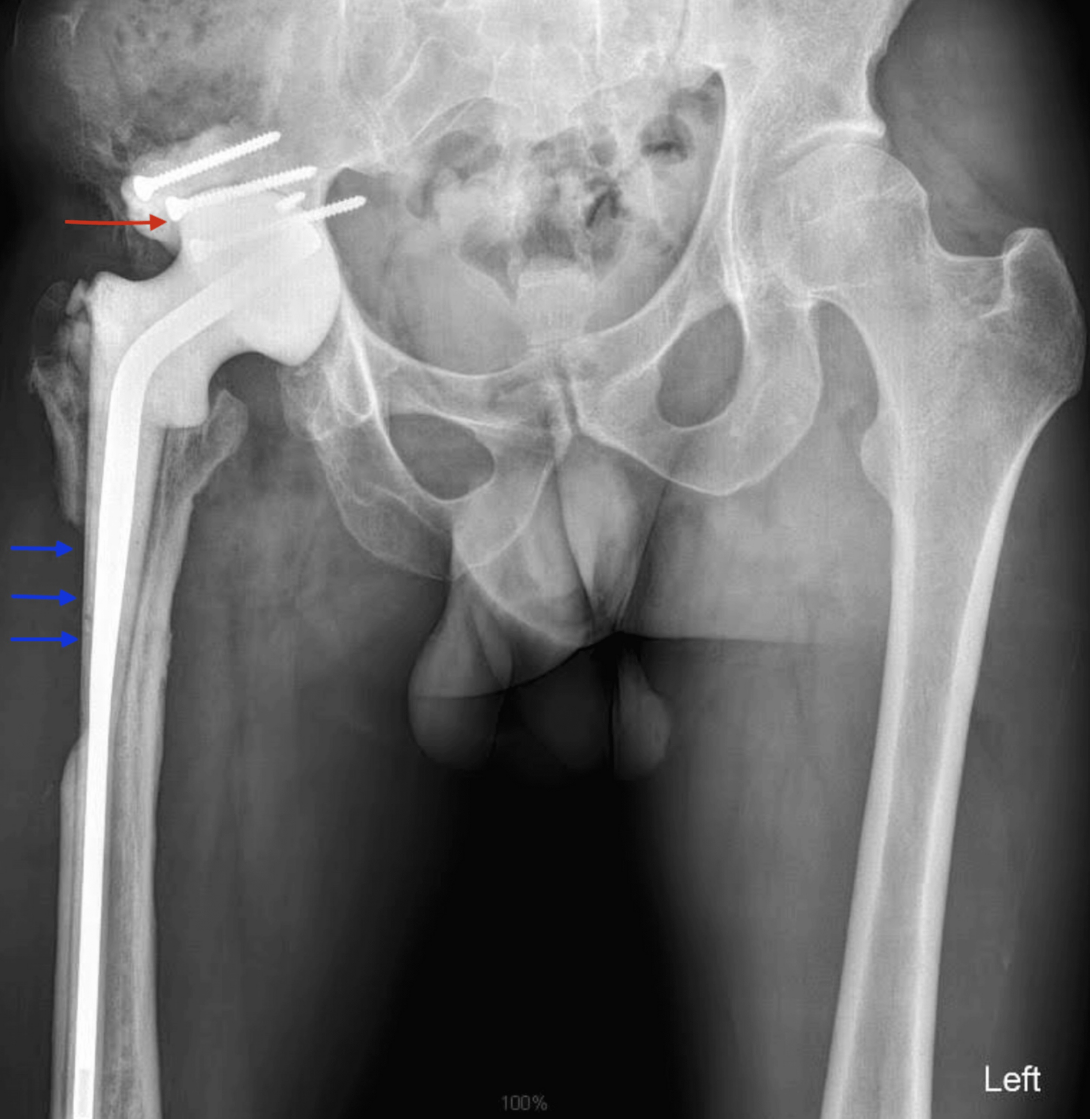
Postoperative X-ray (second spacer exchange) showing bone defects in the acetabulum (red arrow) and proximal femur (blue arrows).

Following the surgical procedure, the C-reactive protein levels remained stable but above the established threshold. The fistula was surgically excised, and the operative wound exhibited primary healing. Subsequently, the patient was discharged. Three months postoperatively, the patient underwent a third revision due to the development of a new fistula. An additional spacer was implanted. Intraoperative samples again revealed the presence of *P. stuartii*, and one sample demonstrated the presence of methicillin-resistant *Staphylococcus epidermidis *(MRSE). The bone quality was significantly compromised, with extensive bone defects observed in both the acetabulum and proximal femur (Figure 4).

**Figure 5:**
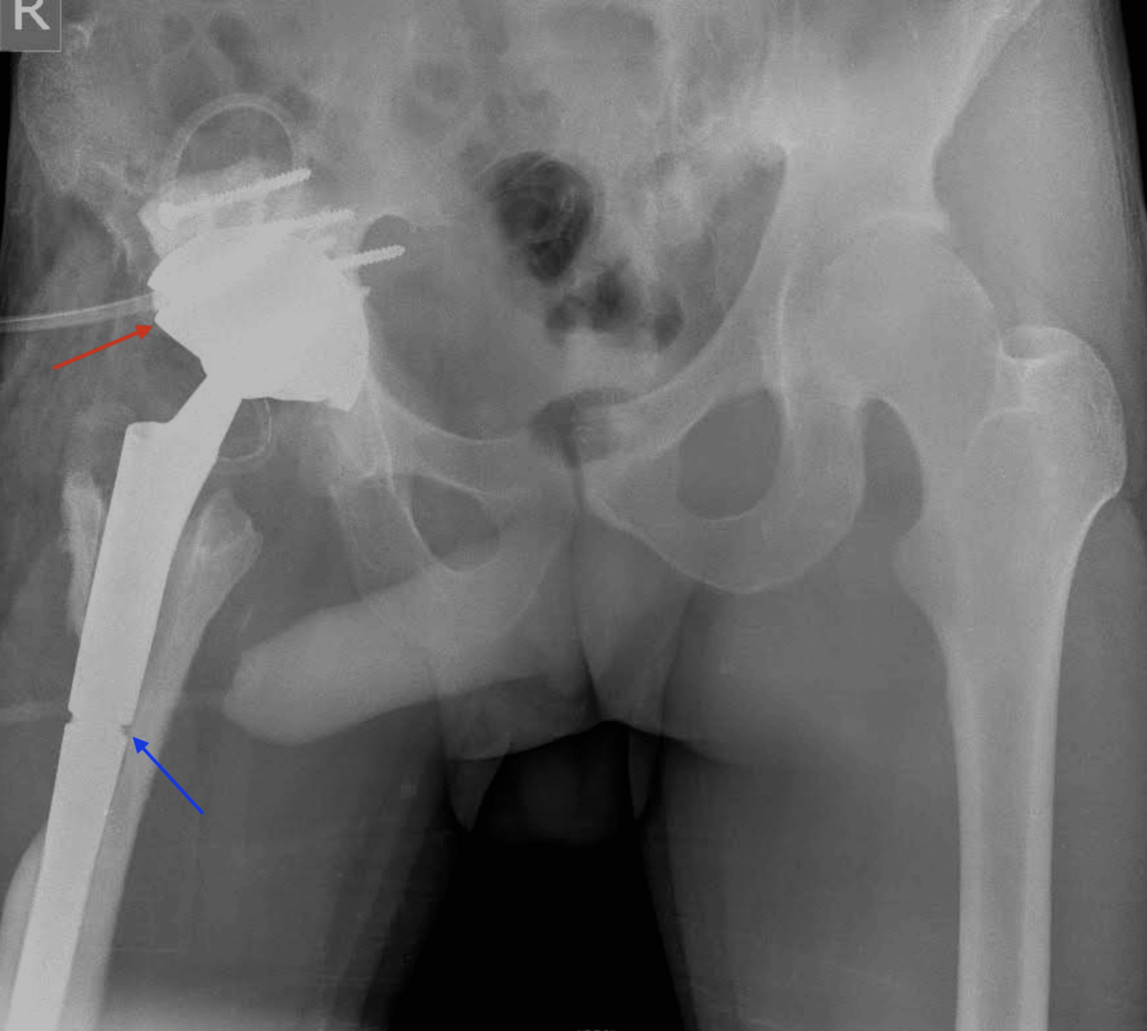
Postoperative X-ray showing a modular revision hip system (blue arrow) with a dual-mobility cup (red arrow).

Following extended antibiotic therapy with meropenem administered via an infusion pump thrice daily for eight hours in conjunction with Tygacil (50mg) twice daily, the patient exhibited rapid recovery. The rehabilitation process progressed satisfactorily, and the patient regained ambulatory function with the spacer. After 10 weeks, the patient was readmitted for the final procedure. Due to bone loss and soft-tissue insufficiency, a modular revision hip system with a duo-mobile cup was implanted (Figure 5).

Five microbiological samples were obtained, none of which indicated infection. The same antibiotic regimen was administered for 14 days. Post-discharge, oral levofloxacin was prescribed for a duration of two months. After a two-year follow-up period, the patient has not manifested any signs of recurrent infection. The Harris Hip Score (HHS) upon initial presentation was 61, and two years post-final surgery, it has improved to 88.

## Discussion

The genus *Providencia* is a urease-producing gram-negative bacillus of the family *Enterobacteriaceae* including *P. stuartii*, primarily associated with urinary tract infections, infections in immunocompromised patients, and patients with diabetes [6-9]. Globally, the prevalence of *P. stuartii* varies based on geographical location, healthcare settings, and patient populations. In hospital settings, studies have shown that *Providencia* is responsible for a small percentage of nosocomial infections, often less than 1% of total infections. Their prevalence may be significantly higher in settings where patients have prolonged hospital stays, are on catheters, or have received antibiotic treatments that disrupt normal flora [10].

*P. stuartii* infrequently causes PJIs. To the best of our knowledge, this is the first case report in the literature of *P. stuartii *causing infection after total hip replacement. In a review from 2019, Fantoni et al. reported two cases of *P. stuartii* in a cohort of 82 patients with PJI in an Italian institute. No information regarding the specific treatment and outcomes of these patients was provided [11].

In the present case, the pathogenesis of the infection remains unclear. There is no information available about the microorganism, which caused the early infection and septic condition. The positive cultures were reported after the total hip replacement, suggesting a potential hematogenous inoculation. Furthermore, *P. stuartii* and *P. retggeri* are reported to be the most common causes of catheter-associated urinary tract infections [6].

*Enterobacteriaceae* are typically not associated with chronic PJI, possibly due to a reduced capacity, in comparison with staphylococci and *P. aeruginosa*, to survive and establish mechanisms of persistence in vivo [12]. The case presented herein exemplifies a chronic PJI. The observed fistula is recognized as a risk factor for polymicrobial infection [13]. This may be the reason why *P. stuartii *has caused chronic PJI in this instance.

The treatment options are limited as the presence of a fistula and the multidrug-resistant organism are contraindications for a one-stage procedure [14]. Furthermore, according to the literature, the debridement, antibiotics, and implant retention (DAIR) procedures demonstrate superior outcomes in acute PJI, compared to chronic infections [15]. The selection of an appropriate surgical strategy is critical. Current guidelines suggest that chronic infections with completed biofilm formation or cases of poor soft tissue condition should undergo complete prosthesis removal and/or exchange, with DAIR being an option in acute cases [16]. The spacer exchange protocol was implemented in this case. The atypical pathogen and the multiresistant antibiogram were responsible for the persistence of the infection after the first spacer implantation. Poor outcomes with this procedure are reported in the literature. The second surgery is invariably associated with higher mortality risk, bone loss, and soft-tissue complications [17,18]. However, the recurrence of infection is most frequently observed in elderly and immunocompromised patients with diminished reparative capacity. The double-exchange protocol can be a viable option for younger patients where infection eradication is of paramount importance.

PJI eradication can only be achieved through the combination of an appropriate surgical strategy and adequate antimicrobial therapy. In this case, the prolonged administration of meropenem via an inclusion pump was essential. A meta-analysis conducted by Yu et al. demonstrates that meropenem exhibits time-dependent antimicrobial activity, and prolonged infusion of meropenem can achieve superior pharmacodynamic targets compared to intermittent bolus administration [19]. However, recent publications from Japan report the emergence of carbapenem-resistant *P. stuartii *[20].

## Conclusions

PJIs caused by multiresistant gram-negative bacteria present significant challenges in both diagnostics and treatment. Our approach, which includes the spacer exchange protocol and prolonged antibiotic therapy utilizing an infusion pump, has shown promise in managing this complex infection. The involvement of a specialized multidisciplinary team is critical, as it allows for a comprehensive evaluation and collaborative decision-making tailored to the unique needs of each patient. However, it is essential to acknowledge the limitations inherent in this case report. Our observations stem from a single patient, which restricts the generalizability of the findings. While the strategies employed were effective in this instance, they may not yield the same outcomes in a broader population or different clinical contexts. Further studies involving larger cohorts are necessary to validate these approaches and establish standardized protocols for managing PJI due to multiresistant gram-negative bacteria. By addressing these limitations and expanding our research, we can enhance our understanding of the complexities involved in treating PJIs and potentially improve patient outcomes in future cases. Continued collaboration among healthcare professionals is vital to refine and optimize treatment strategies, ensuring that we are equipped to tackle the challenges posed by these opportunistic infections.
